# Boston Milk

**Published:** 1871-05

**Authors:** 


Boston Milk.—According to the analyses of twenty samples of adulterated milk by Mr. J. F. Babcock, for the Inspector, the only adulterant found, save a little salt or caramel, was water, the average quantity added being more than thirty-two per cent.

